# Supplementary material for: ProteinShader: illustrative rendering of macromolecules
Source: BMC Struct Biol. 2009 Mar 30;9:19. doi: 10.1186/1472-6807-9-19 (PMC2672931; doi:10.1186/1472-6807-9-19)
Supplement: Additional file 1 — ProteinShader program without source code. This compressed file contains the complete ProteinShader program including associated libraries, but no source code. A README.txt file gives an overview of the ProteinShader distribution, and the index.html file in the help subdirectory has directions on getting started with the program as well as a set of tutorials. [file 1472-6807-9-19-S1.zip › ProteinShader-beta-0_9_4-binary/help/api/org/proteinshader/structure/visitor/LoopGeneratorVisitor.html]

LoopGeneratorVisitor (ProteinShader API)


|  |  |  |
| --- | --- | --- |
| **Overview** \| **Package** \| **Class** \| **Use** \| **Tree** \| **Deprecated** \| **Index** \| **Help** | |  |
| **PREV CLASS**   **NEXT CLASS** | **FRAMES**    **NO FRAMES**     **All Classes** |  |
| SUMMARY: NESTED \| FIELD \| CONSTR \| METHOD | DETAIL: FIELD \| CONSTR \| METHOD |  |


---


## org.proteinshader.structure.visitor Class LoopGeneratorVisitor

```
java.lang.Object
  org.proteinshader.structure.visitor.Visitor
      org.proteinshader.structure.visitor.LoopGeneratorVisitor
```

---

``` public class LoopGeneratorVisitor extends Visitor ```

Generates Loop objects from any AminoAcids of a Chain
that do not already belong to a Helix or BetaStrand.
All Helix and BetaStrand objects should have already been added to
the Structure before this LoopGeneratorVisitor is used. Whenever
a Region is successfully created, each AminoAcid that belongs to
the Region is marked with the regionID and Region type (LOOP, HELIX,
or BETA\_STRAND). When this Visitor is used, any AminoAcids that are
not already assigned to a Region object will be used to create Loop
objects.

---

**Constructor Summary**

`LoopGeneratorVisitor()`
  
          Constructs a LoopGeneratorVisitor.


| **Method Summary** | |
| --- | --- |
| `void` | `visit(AminoAcid aminoAcid)`             Checks whether a start AminoAcid and an end AminoAcid for a Loop have been found, and creates a new Loop as needed. |
| `void` | `visit(Chain chain)`             Generates Loop objects from AminoAcids that do not already belong to any Region. |
| `void` | `visit(Model model)`             Generates all Loop objects needed by the Model. |

**Methods inherited from class org.proteinshader.structure.visitor.Visitor**

`clear, includeAAHetAndWater, setDebug, setMode, setMode, setMode, setMode, setMode, setMode, setVisitSegments, visit, visit, visit, visit, visit, visit, visit, visit, visit`

**Methods inherited from class java.lang.Object**

`clone, equals, finalize, getClass, hashCode, notify, notifyAll, toString, wait, wait, wait`

**Constructor Detail**

### LoopGeneratorVisitor

```
public LoopGeneratorVisitor()
```

:   Constructs a LoopGeneratorVisitor.


**Method Detail**

### visit

```
public void visit(Model model)
           throws VisitorException
```

:   Generates all Loop objects needed by the Model.

    :   **Overrides:**: `visit` in class `Visitor`
    :   **Parameters:**: `model` - the Model to generate Loops for. **Throws:**: `VisitorException` - if an error occurs while generating Bonds.

---


### visit

```
public void visit(Chain chain)
           throws VisitorException
```

:   Generates Loop objects from AminoAcids that do not already
    belong to any Region.

    :   **Overrides:**: `visit` in class `Visitor`
    :   **Parameters:**: `chain` - the Chain to generate Loops for. **Throws:**: `VisitorException` - if an error occurs while generating Loops.

---


### visit

```
public void visit(AminoAcid aminoAcid)
           throws VisitorException
```

:   Checks whether a start AminoAcid and an end AminoAcid for a Loop
    have been found, and creates a new Loop as needed. Any AminoAcids
    that already belong to a Helix or BetaStrand should be marked such
    that aminoAcid.getRegionType() will be non-null.

    :   **Overrides:**: `visit` in class `Visitor`
    :   **Parameters:**: `aminoAcid` - the AminoAcid to test. **Throws:**: `VisitorException` - if an error occurs while generating Loops.


---


|  |  |  |
| --- | --- | --- |
| **Overview** \| **Package** \| **Class** \| **Use** \| **Tree** \| **Deprecated** \| **Index** \| **Help** | |  |
| **PREV CLASS**   **NEXT CLASS** | **FRAMES**    **NO FRAMES**     **All Classes** |  |
| SUMMARY: NESTED \| FIELD \| CONSTR \| METHOD | DETAIL: FIELD \| CONSTR \| METHOD |  |


---

# *Copyright © 2007-2008*
